# Supplementary material for: Modeling glioblastoma heterogeneity as a dynamic network of cell states
Source: Mol Syst Biol. 2021 Sep 16;17(9):e10105. doi: 10.15252/msb.202010105 (PMC8444284; doi:10.15252/msb.202010105)
Supplement: Supplementary file 5 — Source Data for Figure 3 [file MSB-17-e10105-s001.zip › Figure3A_sourcedata/GSEA_3065/hallmarks_state5.GseaPreranked.1623416302988/HALLMARK_ALLOGRAFT_REJECTION.html]

Details for gene set HALLMARK\_ALLOGRAFT\_REJECTION[GSEA]

|  || Dataset | state5 |
| Phenotype | NoPhenotypeAvailable |
| Upregulated in class | na\_neg |
| GeneSet | HALLMARK\_ALLOGRAFT\_REJECTION |
| Enrichment Score (ES) | -0.36703303 |
| Normalized Enrichment Score (NES) | -1.180608 |
| Nominal p-value | 0.180593 |
| FDR q-value | 0.39322668 |
| FWER p-Value | 1.0 |
Table: GSEA Results Summary

  

Fig 1: Enrichment plot: HALLMARK\_ALLOGRAFT\_REJECTION      
 Profile of the Running ES Score & Positions of GeneSet Members on the Rank Ordered List

  

| PROBE | GENE SYMBOL | GENE\_TITLE | RANK IN GENE LIST | RANK METRIC SCORE | RUNNING ES | CORE ENRICHMENT || 1 | B2M |  |  | 61 | 0.601 | 0.0364 | No |
| 2 | TIMP1 |  |  | 109 | 0.518 | 0.0683 | No |
| 3 | CD47 |  |  | 121 | 0.507 | 0.1036 | No |
| 4 | DEGS1 |  |  | 467 | 0.321 | 0.0880 | No |
| 5 | BRCA1 |  |  | 522 | 0.305 | 0.1039 | No |
| 6 | THY1 |  |  | 546 | 0.299 | 0.1228 | No |
| 7 | F2R |  |  | 568 | 0.293 | 0.1415 | No |
| 8 | IFNGR1 |  |  | 983 | 0.213 | 0.1104 | No |
| 9 | GALNT1 |  |  | 1009 | 0.210 | 0.1227 | No |
| 10 | TAPBP |  |  | 1047 | 0.204 | 0.1332 | No |
| 11 | TAP1 |  |  | 1077 | 0.202 | 0.1445 | No |
| 12 | IL27RA |  |  | 1227 | 0.182 | 0.1408 | No |
| 13 | GLMN |  |  | 1375 | 0.164 | 0.1361 | No |
| 14 | EGFR |  |  | 1547 | 0.147 | 0.1275 | No |
| 15 | IKBKB |  |  | 1775 | 0.128 | 0.1113 | No |
| 16 | EIF4G3 |  |  | 1810 | 0.126 | 0.1166 | No |
| 17 | TGFB2 |  |  | 2112 | 0.104 | 0.0903 | No |
| 18 | IFNAR2 |  |  | 2259 | 0.094 | 0.0808 | No |
| 19 | NCK1 |  |  | 2405 | 0.085 | 0.0706 | No |
| 20 | CSF1 |  |  | 2643 | 0.071 | 0.0492 | No |
| 21 | ACVR2A |  |  | 2674 | 0.070 | 0.0508 | No |
| 22 | HDAC9 |  |  | 3051 | 0.049 | 0.0122 | No |
| 23 | TGFB1 |  |  | 3503 | 0.027 | -0.0364 | No |
| 24 | MAP3K7 |  |  | 3835 | 0.014 | -0.0724 | No |
| 25 | TAP2 |  |  | 3919 | 0.011 | -0.0810 | No |
| 26 | TRAF2 |  |  | 4326 | -0.007 | -0.1260 | No |
| 27 | CCND2 |  |  | 4539 | -0.016 | -0.1486 | No |
| 28 | SOCS5 |  |  | 4810 | -0.026 | -0.1770 | No |
| 29 | CD74 |  |  | 5224 | -0.041 | -0.2203 | No |
| 30 | ABI1 |  |  | 5375 | -0.048 | -0.2337 | No |
| 31 | BCL10 |  |  | 5406 | -0.049 | -0.2335 | No |
| 32 | APBB1 |  |  | 5504 | -0.053 | -0.2406 | No |
| 33 | IRF7 |  |  | 5524 | -0.054 | -0.2388 | No |
| 34 | RIPK2 |  |  | 5661 | -0.060 | -0.2497 | No |
| 35 | IL11 |  |  | 5980 | -0.073 | -0.2801 | No |
| 36 | MTIF2 |  |  | 5998 | -0.074 | -0.2767 | No |
| 37 | ABCE1 |  |  | 6018 | -0.075 | -0.2734 | No |
| 38 | STAT1 |  |  | 6029 | -0.076 | -0.2691 | No |
| 39 | CSK |  |  | 6784 | -0.120 | -0.3450 | No |
| 40 | FLNA |  |  | 6856 | -0.126 | -0.3439 | No |
| 41 | IFNGR2 |  |  | 6865 | -0.127 | -0.3357 | No |
| 42 | CCND3 |  |  | 6881 | -0.128 | -0.3282 | No |
| 43 | CAPG |  |  | 6974 | -0.135 | -0.3288 | No |
| 44 | HIF1A |  |  | 6996 | -0.137 | -0.3213 | No |
| 45 | UBE2D1 |  |  | 7178 | -0.154 | -0.3305 | No |
| 46 | BCAT1 |  |  | 7285 | -0.166 | -0.3305 | No |
| 47 | EIF3J |  |  | 7306 | -0.167 | -0.3207 | No |
| 48 | TPD52 |  |  | 7309 | -0.168 | -0.3088 | No |
| 49 | AKT1 |  |  | 7805 | -0.235 | -0.3474 | Yes |
| 50 | EIF3A |  |  | 7813 | -0.236 | -0.3312 | Yes |
| 51 | EIF3D |  |  | 7833 | -0.241 | -0.3160 | Yes |
| 52 | MRPL3 |  |  | 8004 | -0.277 | -0.3152 | Yes |
| 53 | EIF5A |  |  | 8468 | -0.418 | -0.3370 | Yes |
| 54 | UBE2N |  |  | 8529 | -0.447 | -0.3116 | Yes |
| 55 | NME1 |  |  | 8573 | -0.471 | -0.2825 | Yes |
| 56 | NPM1 |  |  | 8706 | -0.566 | -0.2566 | Yes |
| 57 | RPS9 |  |  | 8807 | -0.656 | -0.2207 | Yes |
| 58 | RPL39 |  |  | 8893 | -0.763 | -0.1753 | Yes |
| 59 | RPL9 |  |  | 8918 | -0.813 | -0.1195 | Yes |
| 60 | RPS19 |  |  | 8941 | -0.855 | -0.0605 | Yes |
| 61 | RPS3A |  |  | 8957 | -0.906 | 0.0030 | Yes |
Table: GSEA details [plain text format]

  

Fig 2: HALLMARK\_ALLOGRAFT\_REJECTION: Random ES distribution      
 Gene set null distribution of ES for **HALLMARK\_ALLOGRAFT\_REJECTION**
